# 2-Isopropyl-5-methyl­cyclo­hexyl *N*-cyclo­hexyl-*P*-phenyl­phospho­namidate

**DOI:** 10.1107/S1600536810032319

**Published:** 2010-08-18

**Authors:** Fan-Jie Meng, Hao Xu, Li-Juan Liu, Daqi Wang, Chang-Qiu Zhao

**Affiliations:** a College of Chemistry and Chemical Engineering, Liaocheng University, Shandong 252059, People’s Republic of China

## Abstract

The title compound, C_22_H_36_NO_2_P, features a P atom bonded to a phenyl ring, a cyclo­hexyl­amine unit and the O atom of a menthyl group. In the crystal structure, inter­molecular N—H⋯O hydrogen bonds connect mol­ecules into a one-dimensional chain in the *b* direction.

## Related literature

For the general synthesis of phospho­rus–amine compounds, see: Steinberg (1950 ▶); Benamer *et al.* (2010 ▶). For the structures of related phospho­rus–amine compounds, see: Balakrishna *et al.* (2001 ▶).
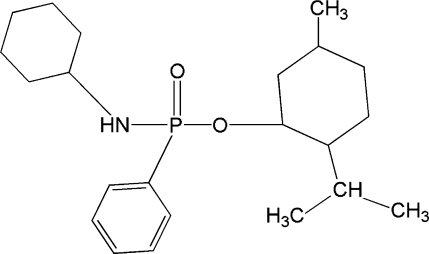


## Experimental

### 

#### Crystal data

C_22_H_36_NO_2_P
*M* *_r_* = 377.49
Orthorhombic, 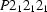

*a* = 10.0205 (10) Å
*b* = 10.4317 (11) Å
*c* = 22.101 (2) Å
*V* = 2310.2 (4) Å^3^
*Z* = 4
Mo *K*α radiation
μ = 0.13 mm^−1^
*T* = 298 K
0.40 × 0.14 × 0.07 mm

#### Data collection

Bruker SMART CCD area-detector diffractometer
Absorption correction: multi-scan (*SADABS*; Sheldrick, 1996 ▶) *T*_min_ = 0.949, *T*_max_ = 0.991
11780 measured reflections
4064 independent reflections
2537 reflections with *I* > 2σ(*I*)
*R*_int_ = 0.061

#### Refinement

*R*[*F* ^2^ > 2σ(*F* ^2^)] = 0.044
*wR*(*F* ^2^) = 0.083
*S* = 1.00
4064 reflections
238 parameters
H-atom parameters constrained
Δρ_max_ = 0.17 e Å^−3^
Δρ_min_ = −0.21 e Å^−3^
Absolute structure: Flack (1983 ▶), 1727 Friedel pairs
Flack parameter: −0.01 (11)

### 

Data collection: *SMART* (Siemens, 1996 ▶); cell refinement: *SAINT* (Siemens, 1996 ▶); data reduction: *SAINT*; program(s) used to solve structure: *SHELXS97* (Sheldrick, 2008 ▶); program(s) used to refine structure: *SHELXL97* (Sheldrick, 2008 ▶); molecular graphics: *SHELXTL* (Sheldrick, 2008 ▶); software used to prepare material for publication: *SHELXTL*.

## Supplementary Material

Crystal structure: contains datablocks I, global. DOI: 10.1107/S1600536810032319/vm2032sup1.cif

Structure factors: contains datablocks I. DOI: 10.1107/S1600536810032319/vm2032Isup2.hkl

Additional supplementary materials:  crystallographic information; 3D view; checkCIF report

## Figures and Tables

**Table 1:** Hydrogen-bond geometry (Å, °)

*D*—H⋯*A*	*D*—H	H⋯*A*	*D*⋯*A*	*D*—H⋯*A*
N1—H1⋯O2^i^	0.86	2.15	2.969 (3)	160

Symmetry code: (i) 
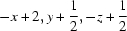
.

**Fig. 1.**
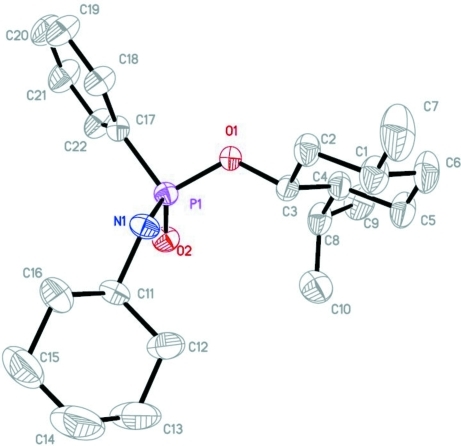
The molecular structure of the compound. H atoms have been omitted for clarity.

**Fig. 2.**
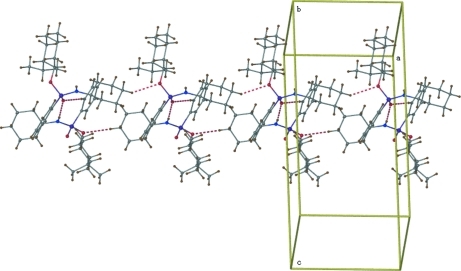
A view of the one-dimensional chain structure formed by N—H···O interactions in the title compound.

## supplementary crystallographic information

### Comment

The molecular structure of the P-chiral title compound consists of an *O*-menthyl phenylphosphinate core and cyclohexylamine (Steinberg, 1950). The absolute configuration of the central P atom is *S* and the four groups around the central P atom form an irregular tetrahedron (Benamer *et al.*, 2010). In the crystal structure intermolecular N—H···O hydrogen bonds connect molecules into a one-dimensional chain in the b-direction (Table 1, Fig. 2).

### Experimental

Carbon tetrachloride was added to a solution of (*R*_p_)-*O*-menthyl-phenyl phosphonothioate dissolved in dry ether and cyclohexylamine. The reaction mixture was stirred for 38 h at room temperature. A single crystal of the title compound suitable for X-ray diffraction was obtained by slow evaporation of an ether solution of the title compound.

### Refinement

The imino H atom was located in a differece Fourier map and refined isotropically, with the N—H distance restrained to 0.86 Å. Other H atoms attached to C atoms were fixed geometrically and treated as riding with C—H = 0.93–0.98 Å, with *U*_iso_(H) = 1.5 *U*_eq_(methyl) and *U*_iso_(H) = 1.2 *U*_eq_(C) for all other H atoms.

### Crystal data

**Table:**

C_22_H_36_NO_2_P	*F*(000) = 824
*M**_r_* = 377.49	*D*_x_ = 1.085 Mg m^−^^3^
Orthorhombic, *P*2_1_2_1_2_1_	Mo *K*α radiation, λ = 0.71073 Å
Hall symbol: P 2ac 2ab	Cell parameters from 2271 reflections
*a* = 10.0205 (10) Å	θ = 3.0–25.0°
*b* = 10.4317 (11) Å	µ = 0.13 mm^−^^1^
*c* = 22.101 (2) Å	*T* = 298 K
*V* = 2310.2 (4) Å^3^	Block, colourless
*Z* = 4	0.40 × 0.14 × 0.07 mm

### Data collection

**Table:**

Bruker SMART CCD area-detector diffractometer	4064 independent reflections
Radiation source: fine-focus sealed tube	2537 reflections with *I* > 2σ(*I*)
graphite	*R*_int_ = 0.061
φ and ω scans	θ_max_ = 25.0°, θ_min_ = 1.8°
Absorption correction: multi-scan (*SADABS*; Sheldrick, 1996)	*h* = −11→11
*T*_min_ = 0.949, *T*_max_ = 0.991	*k* = −11→12
11780 measured reflections	*l* = −26→23

### Refinement

**Table:**

Refinement on *F*^2^	Secondary atom site location: difference Fourier map
Least-squares matrix: full	Hydrogen site location: inferred from neighbouring sites
*R*[*F*^2^ > 2σ(*F*^2^)] = 0.044	H-atom parameters constrained
*wR*(*F*^2^) = 0.083	*w* = 1/[σ^2^(*F*_o_^2^) + (0.0224*P*)^2^] where *P* = (*F*_o_^2^ + 2*F*_c_^2^)/3
*S* = 1.00	(Δ/σ)_max_ = 0.001
4064 reflections	Δρ_max_ = 0.17 e Å^−^^3^
238 parameters	Δρ_min_ = −0.21 e Å^−^^3^
0 restraints	Absolute structure: Flack (1983), with 1727 Friedel pairs [Please check]
Primary atom site location: structure-invariant direct methods	Flack parameter: −0.01 (11)

### Special details

**Table:**

Geometry. All e.s.d.'s (except the e.s.d. in the dihedral angle between two l.s. planes) are estimated using the full covariance matrix. The cell e.s.d.'s are taken into account individually in the estimation of e.s.d.'s in distances, angles and torsion angles; correlations between e.s.d.'s in cell parameters are only used when they are defined by crystal symmetry. An approximate (isotropic) treatment of cell e.s.d.'s is used for estimating e.s.d.'s involving l.s. planes.
Refinement. Refinement of *F*^2^ against ALL reflections. The weighted *R*-factor *wR* and goodness of fit *S* are based on *F*^2^, conventional *R*-factors *R* are based on *F*, with *F* set to zero for negative *F*^2^. The threshold expression of *F*^2^ > σ(*F*^2^) is used only for calculating *R*-factors(gt) *etc*. and is not relevant to the choice of reflections for refinement. *R*-factors based on *F*^2^ are statistically about twice as large as those based on *F*, and *R*- factors based on ALL data will be even larger.

### Fractional atomic coordinates and isotropic or equivalent isotropic displacement parameters (Å^2^)

**Table:**

	*x*	*y*	*z*	*U*_iso_*/*U*_eq_	
N1	1.06771 (19)	0.23385 (19)	0.24171 (9)	0.0499 (6)	
H1	1.0595	0.3159	0.2405	0.060*	
O1	0.83345 (16)	0.21330 (17)	0.19514 (8)	0.0453 (5)	
O2	0.96301 (17)	0.01022 (17)	0.22946 (8)	0.0536 (5)	
P1	0.93290 (8)	0.14723 (7)	0.24088 (3)	0.0429 (2)	
C1	0.8879 (4)	0.4422 (3)	0.06456 (14)	0.0768 (11)	
H1A	0.9796	0.4183	0.0541	0.092*	
C2	0.8593 (3)	0.3933 (3)	0.12811 (12)	0.0606 (9)	
H2A	0.9217	0.4326	0.1561	0.073*	
H2B	0.7701	0.4193	0.1398	0.073*	
C3	0.8704 (3)	0.2485 (3)	0.13321 (12)	0.0481 (8)	
H3	0.9629	0.2226	0.1256	0.058*	
C4	0.7777 (3)	0.1789 (3)	0.08865 (12)	0.0592 (9)	
H4	0.6861	0.2041	0.0987	0.071*	
C5	0.8062 (4)	0.2286 (3)	0.02431 (12)	0.0774 (11)	
H5A	0.8956	0.2033	0.0125	0.093*	
H5B	0.7439	0.1894	−0.0037	0.093*	
C6	0.7939 (4)	0.3748 (3)	0.02013 (14)	0.0895 (12)	
H6A	0.8147	0.4021	−0.0208	0.107*	
H6B	0.7026	0.3997	0.0288	0.107*	
C7	0.8768 (4)	0.5885 (3)	0.06108 (16)	0.1226 (17)	
H7A	0.7875	0.6141	0.0712	0.184*	
H7B	0.8975	0.6165	0.0208	0.184*	
H7C	0.9384	0.6266	0.0891	0.184*	
C8	0.7848 (3)	0.0312 (3)	0.09409 (14)	0.0688 (10)	
H8	0.7767	0.0105	0.1372	0.083*	
C9	0.6676 (4)	−0.0338 (3)	0.06175 (16)	0.0917 (12)	
H9A	0.5853	0.0033	0.0754	0.138*	
H9B	0.6680	−0.1239	0.0708	0.138*	
H9C	0.6762	−0.0217	0.0189	0.138*	
C10	0.9165 (3)	−0.0253 (3)	0.07251 (16)	0.0912 (12)	
H10A	0.9257	−0.0113	0.0298	0.137*	
H10B	0.9178	−0.1157	0.0807	0.137*	
H10C	0.9889	0.0154	0.0934	0.137*	
C11	1.2028 (2)	0.1796 (3)	0.24442 (13)	0.0552 (8)	
H11	1.1964	0.0928	0.2611	0.066*	
C12	1.2647 (3)	0.1704 (4)	0.18205 (14)	0.0822 (11)	
H12A	1.2098	0.1157	0.1568	0.099*	
H12B	1.2665	0.2550	0.1638	0.099*	
C13	1.4051 (4)	0.1172 (5)	0.1840 (2)	0.134 (2)	
H13A	1.4426	0.1165	0.1435	0.161*	
H13B	1.4032	0.0297	0.1989	0.161*	
C14	1.4916 (4)	0.1989 (6)	0.2252 (3)	0.150 (2)	
H14A	1.4984	0.2849	0.2088	0.180*	
H14B	1.5807	0.1628	0.2273	0.180*	
C15	1.4315 (4)	0.2045 (5)	0.2888 (2)	0.1302 (18)	
H15A	1.4860	0.2588	0.3144	0.156*	
H15B	1.4300	0.1192	0.3062	0.156*	
C16	1.2890 (3)	0.2581 (4)	0.28578 (16)	0.0877 (12)	
H16A	1.2505	0.2584	0.3260	0.105*	
H16B	1.2918	0.3459	0.2714	0.105*	
C17	0.8400 (3)	0.1681 (3)	0.30935 (12)	0.0452 (7)	
C18	0.8202 (3)	0.2870 (3)	0.33534 (14)	0.0636 (9)	
H18	0.8568	0.3594	0.3172	0.076*	
C19	0.7467 (3)	0.3002 (3)	0.38791 (15)	0.0776 (11)	
H19	0.7345	0.3810	0.4048	0.093*	
C20	0.6920 (4)	0.1947 (4)	0.41515 (14)	0.0763 (11)	
H20	0.6425	0.2038	0.4505	0.092*	
C21	0.7103 (4)	0.0758 (4)	0.39042 (15)	0.0832 (11)	
H21	0.6733	0.0039	0.4088	0.100*	
C22	0.7844 (3)	0.0634 (3)	0.33780 (14)	0.0673 (10)	
H22	0.7969	−0.0176	0.3213	0.081*	

### Atomic displacement parameters (Å^2^)

**Table:**

	*U*^11^	*U*^22^	*U*^33^	*U*^12^	*U*^13^	*U*^23^
N1	0.0427 (11)	0.0383 (14)	0.0687 (15)	0.0012 (12)	−0.0004 (14)	−0.0012 (12)
O1	0.0465 (11)	0.0518 (13)	0.0377 (11)	0.0046 (10)	−0.0014 (9)	0.0019 (10)
O2	0.0624 (12)	0.0374 (12)	0.0609 (13)	0.0023 (10)	0.0017 (10)	−0.0037 (10)
P1	0.0461 (4)	0.0365 (5)	0.0460 (4)	0.0015 (4)	−0.0010 (4)	−0.0011 (4)
C1	0.115 (3)	0.055 (2)	0.061 (2)	0.002 (2)	−0.006 (2)	0.0105 (17)
C2	0.084 (2)	0.048 (2)	0.050 (2)	0.0012 (18)	−0.0082 (17)	0.0013 (15)
C3	0.0580 (18)	0.047 (2)	0.0387 (18)	0.0040 (16)	0.0001 (15)	0.0008 (14)
C4	0.075 (2)	0.057 (2)	0.0455 (19)	0.006 (2)	−0.0092 (16)	−0.0023 (16)
C5	0.115 (3)	0.067 (3)	0.049 (2)	0.002 (2)	−0.010 (2)	−0.0007 (17)
C6	0.144 (3)	0.071 (3)	0.053 (2)	0.004 (3)	−0.016 (2)	0.0142 (19)
C7	0.217 (5)	0.060 (3)	0.091 (3)	−0.013 (3)	−0.030 (3)	0.029 (2)
C8	0.097 (3)	0.058 (2)	0.052 (2)	−0.007 (2)	−0.012 (2)	−0.0013 (17)
C9	0.113 (3)	0.079 (3)	0.083 (3)	−0.023 (3)	−0.010 (2)	−0.020 (2)
C10	0.106 (3)	0.065 (3)	0.102 (3)	0.017 (3)	−0.020 (3)	−0.023 (2)
C11	0.0372 (15)	0.055 (2)	0.074 (2)	0.0045 (15)	−0.0008 (17)	0.0021 (18)
C12	0.063 (2)	0.093 (3)	0.091 (3)	0.007 (2)	0.0164 (19)	−0.020 (2)
C13	0.071 (3)	0.160 (5)	0.170 (5)	0.018 (3)	0.031 (3)	−0.051 (4)
C14	0.060 (3)	0.183 (6)	0.207 (6)	0.006 (3)	0.011 (3)	−0.030 (5)
C15	0.074 (3)	0.155 (5)	0.162 (5)	0.010 (4)	−0.045 (3)	−0.018 (4)
C16	0.055 (2)	0.114 (3)	0.095 (3)	−0.004 (2)	−0.0194 (19)	−0.008 (2)
C17	0.0514 (17)	0.040 (2)	0.0443 (17)	−0.0032 (17)	−0.0040 (14)	0.0008 (15)
C18	0.080 (2)	0.050 (2)	0.060 (2)	−0.006 (2)	0.0146 (18)	−0.0004 (17)
C19	0.106 (3)	0.064 (3)	0.063 (2)	−0.002 (2)	0.024 (2)	−0.013 (2)
C20	0.105 (3)	0.073 (3)	0.051 (2)	−0.002 (3)	0.026 (2)	0.005 (2)
C21	0.124 (3)	0.064 (3)	0.061 (2)	−0.012 (3)	0.027 (2)	0.010 (2)
C22	0.098 (3)	0.049 (2)	0.055 (2)	−0.008 (2)	0.015 (2)	−0.0009 (16)

### Geometric parameters (Å, °)

**Table:**

N1—C11	1.468 (3)	C9—H9C	0.9600
N1—P1	1.625 (2)	C10—H10A	0.9600
N1—H1	0.8600	C10—H10B	0.9600
O1—C3	1.465 (3)	C10—H10C	0.9600
O1—P1	1.5779 (17)	C11—C16	1.501 (4)
O2—P1	1.4823 (19)	C11—C12	1.515 (4)
P1—C17	1.790 (3)	C11—H11	0.9800
C1—C2	1.522 (4)	C12—C13	1.513 (4)
C1—C6	1.532 (4)	C12—H12A	0.9700
C1—C7	1.532 (4)	C12—H12B	0.9700
C1—H1A	0.9800	C13—C14	1.519 (6)
C2—C3	1.518 (3)	C13—H13A	0.9700
C2—H2A	0.9700	C13—H13B	0.9700
C2—H2B	0.9700	C14—C15	1.529 (6)
C3—C4	1.537 (3)	C14—H14A	0.9700
C3—H3	0.9800	C14—H14B	0.9700
C4—C5	1.540 (4)	C15—C16	1.534 (5)
C4—C8	1.547 (4)	C15—H15A	0.9700
C4—H4	0.9800	C15—H15B	0.9700
C5—C6	1.533 (4)	C16—H16A	0.9700
C5—H5A	0.9700	C16—H16B	0.9700
C5—H5B	0.9700	C17—C22	1.378 (4)
C6—H6A	0.9700	C17—C18	1.382 (4)
C6—H6B	0.9700	C18—C19	1.382 (4)
C7—H7A	0.9600	C18—H18	0.9300
C7—H7B	0.9600	C19—C20	1.370 (4)
C7—H7C	0.9600	C19—H19	0.9300
C8—C10	1.521 (4)	C20—C21	1.367 (4)
C8—C9	1.533 (4)	C20—H20	0.9300
C8—H8	0.9800	C21—C22	1.386 (4)
C9—H9A	0.9600	C21—H21	0.9300
C9—H9B	0.9600	C22—H22	0.9300
			
C11—N1—P1	123.52 (17)	H9A—C9—H9C	109.5
C11—N1—H1	118.2	H9B—C9—H9C	109.5
P1—N1—H1	118.2	C8—C10—H10A	109.5
C3—O1—P1	123.26 (16)	C8—C10—H10B	109.5
O2—P1—O1	116.15 (11)	H10A—C10—H10B	109.5
O2—P1—N1	111.64 (11)	C8—C10—H10C	109.5
O1—P1—N1	106.81 (11)	H10A—C10—H10C	109.5
O2—P1—C17	111.54 (12)	H10B—C10—H10C	109.5
O1—P1—C17	99.19 (11)	N1—C11—C16	110.2 (2)
N1—P1—C17	110.81 (12)	N1—C11—C12	111.4 (2)
C2—C1—C6	108.8 (3)	C16—C11—C12	110.7 (2)
C2—C1—C7	111.5 (3)	N1—C11—H11	108.2
C6—C1—C7	112.4 (3)	C16—C11—H11	108.2
C2—C1—H1A	108.0	C12—C11—H11	108.2
C6—C1—H1A	108.0	C13—C12—C11	112.2 (3)
C7—C1—H1A	108.0	C13—C12—H12A	109.2
C3—C2—C1	112.8 (2)	C11—C12—H12A	109.2
C3—C2—H2A	109.0	C13—C12—H12B	109.2
C1—C2—H2A	109.0	C11—C12—H12B	109.2
C3—C2—H2B	109.0	H12A—C12—H12B	107.9
C1—C2—H2B	109.0	C12—C13—C14	110.0 (4)
H2A—C2—H2B	107.8	C12—C13—H13A	109.7
O1—C3—C2	107.5 (2)	C14—C13—H13A	109.7
O1—C3—C4	109.1 (2)	C12—C13—H13B	109.7
C2—C3—C4	112.2 (2)	C14—C13—H13B	109.7
O1—C3—H3	109.3	H13A—C13—H13B	108.2
C2—C3—H3	109.3	C13—C14—C15	110.3 (4)
C4—C3—H3	109.3	C13—C14—H14A	109.6
C3—C4—C5	108.7 (2)	C15—C14—H14A	109.6
C3—C4—C8	113.1 (2)	C13—C14—H14B	109.6
C5—C4—C8	113.5 (2)	C15—C14—H14B	109.6
C3—C4—H4	107.1	H14A—C14—H14B	108.1
C5—C4—H4	107.1	C14—C15—C16	109.9 (4)
C8—C4—H4	107.1	C14—C15—H15A	109.7
C6—C5—C4	112.1 (3)	C16—C15—H15A	109.7
C6—C5—H5A	109.2	C14—C15—H15B	109.7
C4—C5—H5A	109.2	C16—C15—H15B	109.7
C6—C5—H5B	109.2	H15A—C15—H15B	108.2
C4—C5—H5B	109.2	C11—C16—C15	111.3 (3)
H5A—C5—H5B	107.9	C11—C16—H16A	109.4
C1—C6—C5	111.6 (3)	C15—C16—H16A	109.4
C1—C6—H6A	109.3	C11—C16—H16B	109.4
C5—C6—H6A	109.3	C15—C16—H16B	109.4
C1—C6—H6B	109.3	H16A—C16—H16B	108.0
C5—C6—H6B	109.3	C22—C17—C18	117.6 (3)
H6A—C6—H6B	108.0	C22—C17—P1	120.0 (2)
C1—C7—H7A	109.5	C18—C17—P1	122.4 (2)
C1—C7—H7B	109.5	C17—C18—C19	121.0 (3)
H7A—C7—H7B	109.5	C17—C18—H18	119.5
C1—C7—H7C	109.5	C19—C18—H18	119.5
H7A—C7—H7C	109.5	C20—C19—C18	120.2 (3)
H7B—C7—H7C	109.5	C20—C19—H19	119.9
C10—C8—C9	110.3 (3)	C18—C19—H19	119.9
C10—C8—C4	113.7 (3)	C21—C20—C19	120.0 (3)
C9—C8—C4	111.6 (3)	C21—C20—H20	120.0
C10—C8—H8	106.9	C19—C20—H20	120.0
C9—C8—H8	106.9	C20—C21—C22	119.5 (3)
C4—C8—H8	106.9	C20—C21—H21	120.3
C8—C9—H9A	109.5	C22—C21—H21	120.3
C8—C9—H9B	109.5	C17—C22—C21	121.7 (3)
H9A—C9—H9B	109.5	C17—C22—H22	119.1
C8—C9—H9C	109.5	C21—C22—H22	119.1
			
C3—O1—P1—O2	−73.7 (2)	P1—N1—C11—C16	−139.8 (2)
C3—O1—P1—N1	51.5 (2)	P1—N1—C11—C12	96.9 (3)
C3—O1—P1—C17	166.7 (2)	N1—C11—C12—C13	178.8 (3)
C11—N1—P1—O2	−12.7 (2)	C16—C11—C12—C13	55.9 (4)
C11—N1—P1—O1	−140.6 (2)	C11—C12—C13—C14	−56.8 (5)
C11—N1—P1—C17	112.3 (2)	C12—C13—C14—C15	57.5 (6)
C6—C1—C2—C3	55.9 (4)	C13—C14—C15—C16	−57.7 (6)
C7—C1—C2—C3	−179.6 (3)	N1—C11—C16—C15	−179.2 (3)
P1—O1—C3—C2	−117.1 (2)	C12—C11—C16—C15	−55.6 (4)
P1—O1—C3—C4	121.0 (2)	C14—C15—C16—C11	57.0 (5)
C1—C2—C3—O1	−176.8 (2)	O2—P1—C17—C22	−12.2 (3)
C1—C2—C3—C4	−56.9 (4)	O1—P1—C17—C22	110.8 (2)
O1—C3—C4—C5	173.4 (2)	N1—P1—C17—C22	−137.2 (2)
C2—C3—C4—C5	54.4 (3)	O2—P1—C17—C18	168.3 (2)
O1—C3—C4—C8	−59.6 (3)	O1—P1—C17—C18	−68.8 (3)
C2—C3—C4—C8	−178.6 (3)	N1—P1—C17—C18	43.3 (3)
C3—C4—C5—C6	−55.0 (4)	C22—C17—C18—C19	−0.2 (5)
C8—C4—C5—C6	178.2 (3)	P1—C17—C18—C19	179.3 (2)
C2—C1—C6—C5	−55.9 (4)	C17—C18—C19—C20	−0.1 (5)
C7—C1—C6—C5	−179.9 (3)	C18—C19—C20—C21	0.2 (6)
C4—C5—C6—C1	57.6 (4)	C19—C20—C21—C22	0.0 (6)
C3—C4—C8—C10	−69.3 (3)	C18—C17—C22—C21	0.4 (4)
C5—C4—C8—C10	55.2 (4)	P1—C17—C22—C21	−179.2 (3)
C3—C4—C8—C9	165.2 (2)	C20—C21—C22—C17	−0.3 (5)
C5—C4—C8—C9	−70.4 (4)		

### Hydrogen-bond geometry (Å, °)

**Table:**

*D*—H···*A*	*D*—H	H···*A*	*D*···*A*	*D*—H···*A*
N1—H1···O2^i^	0.86	2.15	2.969 (3)	160

Symmetry codes: (i) −*x*+2, *y*+1/2, −*z*+1/2.
